# Interpreting Therapeutic Effects in Single‐Case Designs: Cautionary Notes on Combined Music Therapy and Pharmacotherapy

**DOI:** 10.1002/ccr3.72906

**Published:** 2026-06-09

**Authors:** Chukwuka Elendu, Ifeanyi M. Nnaemeka, Ebiere O. Osusu, Augustine O. Nnosiri

**Affiliations:** ^1^ Federal University Teaching Hospital Owerri Nigeria; ^2^ Priory Hospital Stockton Hall York UK; ^3^ Calderdale and Huddersfield NHS Foundation Trust Huddersfield UK; ^4^ Cygnet Hospital Oldbury Oldbury UK

Dear Editor,

1

We read with considerable interest the recently published case report titled “Music Therapy and Pharmacotherapy as a Combination Treatment: A Case of Periodic Depression in Comorbidity With Subthreshold Autism” [1], which describes a 44‐year‐old patient with recurrent major depressive disorder and traits suggestive of subthreshold autism spectrum disorder who experienced symptomatic improvement following a treatment regimen combining pharmacotherapy and music therapy. The authors provide an informative narrative of a complex clinical trajectory and highlight the potential value of music therapy as an adjunctive modality in patients whose depressive symptoms may be intertwined with atypical emotional processing [2, 3]. Importantly, the combined use of pharmacotherapy and music therapy in this case reflects the realities of individualized clinical practice and offers meaningful teaching points regarding multimodal management strategies in complex psychiatric presentations. Although the relative contribution of each intervention cannot be fully distinguished within a single‐case framework, the observed clinical improvement nonetheless provides valuable insight into how integrative therapeutic approaches may benefit selected patients in real‐world settings [4].

The patient described had a long‐standing history of recurrent depressive episodes beginning in early adulthood and had undergone multiple pharmacological trials prior to the introduction of music therapy. The clinical narrative suggests that the combination of antidepressant therapy with individualized music therapy sessions contributed to improvements in emotional regulation, relational functioning, and depressive symptom burden. Although such observations are clinically intriguing, the interpretation of causality in single‐case designs requires careful methodological scrutiny [4]. In situations where several interventions are implemented simultaneously or sequentially within overlapping time frames, distinguishing the relative contribution of each therapeutic component becomes inherently difficult.

Single‐case studies have historically played a valuable role in generating clinical hypotheses, particularly in emerging or interdisciplinary treatment domains [4]. However, their interpretive strength depends heavily on methodological rigor, including clearly defined intervention phases, consistent measurement of clinical outcomes, and efforts to control for potential confounders [4]. In the present report, pharmacological management and music therapy appear to have been implemented during overlapping periods of treatment, making it challenging to determine whether improvements in depressive symptoms were attributable to the music therapy intervention itself, medication adjustments, natural fluctuation of the disorder, or an interaction among these factors (Table 1). This issue is especially relevant in mood disorders, where symptom trajectories are often variable and influenced by psychosocial context, environmental stressors, and spontaneous remission patterns [5]. Furthermore, the report does not describe the use of standardized longitudinal symptom rating instruments—such as validated depression severity scales—to objectively quantify clinical changes over time. The absence of structured outcome measurement limits the ability to evaluate the magnitude and temporal pattern of symptom improvement.

**TABLE 1:** Factors influencing the interpretation of therapeutic outcomes in the reported case.

Domain	Observation in the case report	Potential implication for interpretation
Concurrent interventions	Music therapy was administered during periods of ongoing pharmacological adjustments.	Makes it difficult to distinguish whether symptom improvement resulted from medication effects, music therapy, or their interaction.
Pharmacotherapy changes	Multiple antidepressants were trialed before stabilization on citalopram and nortriptyline.	Clinical improvement may reflect medication optimization rather than the adjunctive effect of music therapy.
Natural course of depression	Recurrent major depressive disorder often follows fluctuating trajectories with spontaneous partial remissions.	Observed improvement may partly reflect natural illness variability rather than treatment‐specific effects.
Outcome measurement	No standardized longitudinal symptom rating scales were reported.	Limits objective assessment of treatment response and the magnitude of symptom change over time.
Expectancy and engagement effects	The patient reportedly had a long‐standing affinity for music and entered therapy with a positive attitude.	Positive expectations and motivation may enhance perceived therapeutic benefits independent of treatment efficacy.
Therapeutic environment	Regular clinical follow‐up and supportive therapist interaction occurred during treatment.	Nonspecific therapeutic factors such as therapeutic alliance, structure, and social support may contribute to clinical improvement.
Experimental structure	The report did not employ structured single‐case experimental designs (e.g., baseline phases, A–B–A reversal, multiple baseline designs).	Absence of experimental control reduces the ability to infer causal relationships between intervention and outcome.

The difficulty of isolating treatment effects becomes even more pronounced in cases involving recurrent depressive disorder. Major depressive disorder is characterized by episodic fluctuations in symptom severity, and remission may occur independently of specific therapeutic interventions [5]. Indeed, longitudinal research demonstrates that depressive episodes frequently show partial improvement over time, even in the absence of structured intervention. Consequently, attributing the clinical improvement observed in the reported patient solely to a particular therapeutic modality without clearly defined and controlled observation periods during follow‐up may risk overstating the efficacy of that intervention [4].

Another important methodological consideration relates to the interpretation of emotional and behavioral changes observed during music therapy sessions. The authors describe how the patient engaged in musical improvisation, emotional expression through instrumental play, and guided exploration of affective states, which reportedly facilitated improved emotional awareness and regulation. While these observations are consistent with theoretical models of expressive therapies [2], it remains unclear whether the reported improvements represent durable therapeutic change or context‐specific emotional responses occurring within the therapeutic environment. Psychotherapeutic settings often provide supportive relational structures that themselves can promote symptom relief independent of the specific technique employed. In addition, preexisting familiarity and positive engagement with music may influence an individual's receptivity to music‐based therapeutic interventions and contribute to enhanced subjective therapeutic experiences [6, 7].

Furthermore, the case report emphasizes the potential role of subthreshold autism spectrum traits in shaping the patient's depressive experience. Individuals with autism spectrum conditions frequently exhibit differences in emotional processing, social communication, and sensory integration, which may influence how they engage with therapeutic modalities [8, 9]. Music‐based interventions have been proposed as particularly beneficial in this population because they offer nonverbal channels for emotional expression and interpersonal engagement [10]. However, it is important to recognize that the patient described did not meet the full diagnostic criteria for autism spectrum disorder according to the reported evaluation. Nevertheless, the presence of subthreshold autistic traits may still provide clinically relevant context for understanding the patient's engagement with music‐based therapy and the individualized therapeutic approach described in the case report.

In addition to the interpretive challenges inherent in single‐case methodology, the case highlights an important issue regarding treatment sequencing in complex psychiatric presentations. The patient received numerous pharmacological interventions over the course of treatment, including various antidepressants, mood stabilizers, and adjunctive medications. Ultimately, the combination of citalopram and nortriptyline was reported to provide the most tolerable and effective pharmacological regimen. As pharmacotherapy adjustments and music therapy were administered during overlapping periods of care, the case illustrates the potential clinical value of a multimodal therapeutic approach, although the relative contribution of each intervention cannot be definitively distinguished within the context of a single‐case report.

The therapeutic environment itself may also play a significant role in shaping outcomes in complex psychiatric cases. The report describes an extended treatment period involving regular outpatient follow‐up, supportive therapeutic interactions, and structured engagement with clinicians. Such factors are known to contribute to improvements in depressive symptoms through mechanisms including therapeutic alliance, increased behavioral activation [11], and enhanced social support. In this context, music therapy may have functioned as a structured medium through which these broader therapeutic processes could unfold rather than as the primary mechanism driving clinical improvement.

It is also worth noting that the patient participated in a substantial number of music therapy sessions—45 individual sessions over approximately 18 months. Extended engagement with any therapeutic modality may facilitate gradual changes in emotional awareness, coping strategies, and interpersonal functioning. However, without systematic measurement of symptom trajectories across clearly defined treatment phases, it remains difficult to determine whether the improvements reported were temporally associated with specific therapeutic interventions or whether they emerged gradually through cumulative psychosocial processes. Although the original report was appropriately presented as a clinical case report rather than a single‐case experimental study, the incorporation of structured single‐case design elements—such as baseline observation periods, A–B–A reversal phases, or multiple baseline approaches—in future investigations may further strengthen longitudinal assessment and interpretation of therapeutic outcomes in similar clinical contexts.

Despite these methodological considerations, the case nonetheless contributes meaningfully to ongoing discussions about integrative approaches to depression treatment. The intersection between mood disorders and neurodevelopmental traits represents a clinically important area that remains insufficiently explored. Individuals with autism spectrum characteristics often encounter unique challenges in conventional psychotherapeutic settings, particularly when interventions rely heavily on verbal emotional processing. In such contexts, alternative expressive modalities such as music therapy may offer valuable avenues for engagement and emotional exploration [6, 7].

The neurobiological framework proposed by the authors further highlights the theoretical plausibility of music‐based interventions in psychiatric treatment. Music processing engages multiple neural systems involved in emotion regulation, reward processing, and cognitive integration. Studies in neuroscience have demonstrated that musical improvisation and listening can activate networks linking auditory processing with limbic and prefrontal regions, which are known to play critical roles in mood regulation and emotional expression. Such findings provide a conceptual basis for considering music therapy as a complementary modality within broader treatment strategies for mood disorders.

Importantly, this case report provides valuable clinical insights into the potential role of combined music therapy and pharmacotherapy in managing complex depressive presentations and highlights the relevance of individualized, multimodal therapeutic approaches in real‐world psychiatric care. Future research may build upon the observations and teaching points generated from such case reports by incorporating standardized outcome measures, longitudinal follow‐up, and comparative study designs to further explore the role of music therapy as an adjunctive intervention in depression management [10, 12].

Another promising direction for future research involves examining individual differences that may predict responsiveness to music‐based interventions. Factors such as baseline emotional regulation capacity, sensory sensitivity, and neurodevelopmental traits may influence how individuals engage with music therapy and the degree to which they benefit from it. Identifying such predictors could help clinicians tailor treatment strategies more effectively and determine which patients are most likely to benefit from integrative therapeutic approaches.

The case also demonstrates the importance of interdisciplinary collaboration in the management of complex psychiatric conditions. The integration of pharmacological treatment with psychotherapeutic and expressive modalities reflects a growing recognition that mental health disorders often require multifaceted treatment strategies. In practice, combining biological and psychosocial interventions may enhance patient engagement and address dimensions of experience that might otherwise remain inaccessible through a single therapeutic approach.

In conclusion, combined music therapy and pharmacotherapy may provide meaningful benefits in complex depressive presentations. However, interpretation of therapeutic effects in single‐case settings requires caution because of concurrent interventions and the absence of standardized outcome assessment. Further studies with structured follow‐up may help clarify the role of music therapy in multimodal depression management.

## Author Contributions

**Chukwuka Elendu:** conceptualization, investigation, data curation, methodology, formal analysis, writing – original draft, writing – review and editing, validation, visualization, supervision, project administration. **Ifeanyi M. Nnaemeka:** validation, writing – review and editing. **Ebiere O. Osusu:** validation, writing – review and editing. **Augustine O. Nnosiri:** validation, writing – review and editing.

## Funding

The authors have nothing to report.

## Disclosure

The views expressed in this report are solely those of the authors and do not represent the official positions of any affiliated institutions.

## Ethics Statement

Ethical approval was not required, as this letter does not involve new patient data, human subject research, or original clinical investigation.

## Consent

Written informed consent was not required as this letter does not include new patient data.

## Conflicts of Interest

The authors declare no conflicts of interest.

## Data Availability

Data sharing is not applicable to this article as no datasets were generated or analyzed during this study.
